# Chinese Digital Arm (CDA): A High-Precision Digital Arm for Electrical Stimulation Simulation

**DOI:** 10.3390/bioengineering10030374

**Published:** 2023-03-18

**Authors:** Shuang Zhang, Jiujiang Wang, Yuanyu Yu, Lin Wu, Tao Zhang

**Affiliations:** 1 The School of Artificial Intelligence, Neijiang Normal University, Neijiang 641004, China; 2 The School of Life Science and Technology, University of Electronic Science and Technology of China, Chengdu 610056, China; 3 The NJNU-OMNISKY Smart Medical Engineering Applications Joint Laboratory, Neijiang Normal University, Neijiang 641004, China; 4 The High Field Magnetic Resonance Brain Imaging Laboratory of Sichuan, Chengdu 610056, China; 5 The Sichuan Institute for Brain Science and Brain-Inspired Intelligence, Chengdu 610056, China

**Keywords:** image generation, reconstruction, segmentation, stimulation

## Abstract

To effectively analyze the diffusion and accumulation of signals on the surface and inside the human body under electrical stimulation, we used the gray threshold of the Chinese Digital Human image dataset to segment an arm image and reconstruct the tissue to obtain its three-dimensional cloud point dataset. Finally, a semirefined digital arm entity model with the geometric characteristics of the actual human arm tissue was constructed using reverse engineering technology. Further input of the current signal stimulation under tDCS and tACS with additional analysis of the signal diffusion in the transient mode via model calculation revealed that DC electrical stimulation is likely to cause high-voltage burns. The effective depth achieved using the AC stimulation signal is considerable, and provides reference for the electrical stimulation selection. Simultaneously, in the digital arm model, the signal diffusion and tissue damage inside the arm can be analyzed by changing the field, which provides a theoretical basis for the experimental study of the human body.

## 1. Introduction

With the rapid development of social economy, human lifespan is increasing. Many people suffer from functional neurological diseases, such as insomnia, chronic pain, and depression [1,2]. Functional neuropathy is a disease that poses a threat to human quality of life and health. Presently, neurological diseases are mainly treated using drug [3,4,5] and surgical interventions [6,7,8] to alleviate symptoms. However, prolonged antipsychotic drug use leads to drug resistance, which, in turn complicates downstream patient treatment [4,5]. Furthermore, surgical treatment easily damages other tissues and organs, and requires highly accurate and precise medical diagnosis, and surgical operation, which, in turn, complicates surgery implementation [6,7,8]. Therefore, treatment plans that can minimize psychological and physical injuries of patients, with minor side effects, have been explored extensively. Non-invasive electrical stimulation neuromodulation technology has become an important tool for treating functional neurological diseases owing to its simple operation, absence of side effects, and safety for human tissues [9,10,11,12,13,14,15,16,17].

Non-invasive electrical stimulation technology was first established in 46 AD. Scibonius Largus used a current generated by electric eels to treat a Roman emperor with headache and gout-related disease [9], which represents the first record of use of non-invasive electrical stimulation technology in disease treatment. In 1777, Cavallo published a report on the use of the electrical stimulation technology to treat epilepsy, paralysis, chorea, deafness, blindness, rheumatism, and gland enlargement, consequently expanding the scope of application of electrical stimulation technology in pathological regulation [10].

In 1818, when Andrew Ure used DC electrical stimulation on the chest of hanged prisoners, they observed muscle contraction in the fresh corpses, which led to the conclusion that electrical stimulation could resuscitate patients whose organs were undamaged [11]. Accordingly, electric shock cardiac resuscitation was introduced in modern medicine. In 1870, Fritsch and Hitzig observed the limb activities of dogs under motor cortex stimulation [12], which demonstrated, for the first time, that electrical stimulation could influence the activity of the motor nerves of animals. In 1874, Roberts Bartholow applied electrical stimulation technology to a living person for the first time, inducing a grand epilepsy seizure [13], which further demonstrates that electrical stimulation technology can effectively regulate functional neurological diseases but may also induce new diseases if used improperly. In 1963, Sam Jacobsen proposed the use of electrical stimulation to treat mental diseases and achieved notable results [14], which represents the first report of the use of electrical stimulation to treat mental disease.

In modern medicine, nondestructive electrical stimulation technology is used extensively. In 2008, Chet T. Moritz of the University of Washington applied electrical stimulation on a monkey paralyzed by spinal cord injury, so that the monkey could control a computer cursor and mechanical arm [15], which further suggests that electrical stimulation technology can effectively improve functional injuries of the nervous system. In 2018, three patients with spinal cord injury could walk again following administration of directional spinal cord electrical stimulation, which established a technical framework for the enhancement of recovery of neural function following spinal cord injury [16]. In the same year, the stimulation scheme was reportedly ineffective for walking recovery because the current interfered with the patient’s perception of limb position during stimulation; the short stimulus not only promoted movement but also retained the sensory signals from the legs [17]. According to the results, stimulation precision is crucial for the achievement of appropriate feedback.

The finite element modeling method represents the most direct and effective strategy of providing an accurate electrical stimulation scheme. To minimize modeling difficulty and improve the computing speed of a computer, current neural regulation and source location modeling are typically implemented using simplified models [18,19,20,21]. This leads to substantial discrepancies with the actual human tissue structure, and the calculation results often differ considerably from the actual measurement [18,19]. To this end, some researchers have proposed to use medical images to construct a digital human architecture with human tissue characteristics. The digital human plan developed by the Swiss IT’IS Found [22,23] provided a complete human dataset with an organ segmentation scheme. Subsequently, they developed the Sim4Life software platform, which is suitable for use in a variety of physical fields, including both industrial and research applications. However, the data of the platform cannot be exported, limiting its secondary research and development application.

The application of the Sim4Life software mainly focused on magnetic resonance imaging (MRI) coil design and electromagnetic simulation, and cannot effectively simulate electrical stimulation. Japan National Institute of Informatics (NII) [24], South Korea Electronics and Telecommunications Research Institute (ETRI) [25,26,27,28], United Kingdom (UK) National Radiological Protection Board (NRPB) [29], Germany Munchen, Computer Simulation Technology AG (CST) [29,30,31], the United States Pennstate [32], University of Texas at Austin, and others [30,31,33,34,35,36,37,38], have also developed digital human models; however, due to incomplete public data and rough data segmentation schemes, a large number of singularities have been generated after data segmentation, which complicate reverse engineer modeling of human tissues and organs. The Chinese visible human (CVH) [39,40,41,42,43], developed under the leadership of Zhang Shaoxiang of the Army Medical University (formerly the Third Military Medical University), has high slicing accuracy and complete data. Notably, it can extract nearly 110 tissues/organs in the whole body and 32 tissues/organs (including some nerves) in the head via image gray value segmentation. It provides fundamental data for establishing a high-precision electrical stimulation model of neural regulation.

In the present study, the image gray threshold was used to segment and reconstruct images based on the CVH dataset to study the diffusion of electrical stimulation signals in the human body. Finally, a semirefined Chinese digital arm (CDA) model with the geometric characteristics of the real human arm tissue was constructed via inverse engineering technology. Based on the model, the diffusion of current signal stimulation in the human body under the two stimulation modes, tDCS and tACS, was analyzed. Compared with the multilayer uniform cylinder built by Wegmueller [44] and Song [45], the model can more realistically represent the uneven distribution of tissues in the human body, and the uneven distribution will directly influence the conduction of current signals in the human body [46]. In addition, a realistic representation, supported by anatomical consideration of the tissue response to electrical stimulation (tDCS [47] and tACS [48,49]) and their mathematical/physical modeling, is lacking. In the present study, the human arm was the research object, and the Chinese digital human image dataset was the modeling data basis. The multimodal image organization and identification provide a theoretical basis for tDCS and tACS research through identification, segmentation, reconstruction, and physical field modeling.

In this study, firstly, we summarize the research progress of the electrical stimulation technology, the digital arm, and analyze the feasibility of building a CDA using the Chinese Digital Human dataset. Secondly, we present the CDA production process. Thirdly, we introduce the finite element modeling experiment, and analyze the calculation results of two stimulation modes: tDCS and tACS. Finally, we present a technical discussion and summary as per the modeling results.

## 2. Data Preprocessing

### 2.1. Data Foundation

A high-precision digital arm model was constructed based on the CVH image dataset developed by the Army Medical University [37,40,41]. A total of 2518 cross sectional images (slice thickness: 0.10 mm and 0.20 mm for the head, 0.50 mm for the knee joint, and 1.0 mm for other parts) were obtained from a male cadaver with a body length of 172 cm (the maximum extension from head to toe). The slice resolution was 6,291,456 (30,722,048) pixels. The images (*n* = 1756) were extracted as the whole-body data of the digital human, with a sampling interval of ≤1 mm. To build a complete and high-precision arm model, 727 images starting from the shoulder (the first image) were extracted and ending at the finger (the last image) as the fundamental data for CDA 3D reconstruction.

Computerized tomography (CT), MRI, and radiology images were also included in the dataset to obtain accurate tissue parameters; the MRI images of the sample body were scanned in the sagittal plane and coronal plane at 3.0 mm intervals, and the CT dataset was scanned in the sagittal plane at 1.0 mm intervals. In tissue segmentation, the gray value information of tissue images mined from CT, MRI, and radiology images provide the segmentation index information for tissue segmentation.

### 2.2. Tissue Segmentation

The quantized tissue slice images were imported into the image segmentation software Mimics 15.0 (Materialise, Brussels, Belgium), and the grayscale value of the tissue was used as the threshold for implementing segmentation. For major tissues, the splitting method is mainly used to implement segmentation (we first select all tissue thresholds, build the overall tissue segmentation, and remove the background part; then select all parts excluding the background and skin, and build other tissue parts. The skin tissue part can be obtained using Boolean operation on the constructed part), to avoid singular points, and sharp faces. Layer-by-layer stripping was performed until the innermost bone marrow was extracted. The stripped tissue was then built into a tissue cloud point dataset to provide a data foundation for further geometric entity construction required for simulation.

### 2.3. Reconstruction of the Geometric Entities

**Figure 1:**
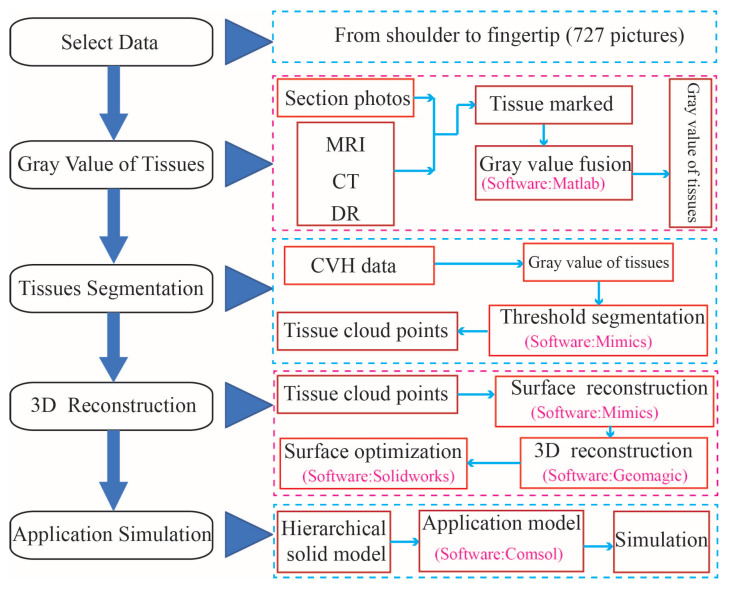
The flowchart for the 3D reconstruction of the Chinese digital arm (CDA)-based Chinese Visible Human (CVH). MRI, magnetic nuclear resonance; CT, computerized tomography; DR, Digital Radiography.

To ensure that the tissue geometric entity is provided for the subsequent tissue modeling, the sharp parts and singular points introduced by the tissue segmentation are removed, as well as to avoid overly fine or complicated singular point segmentation, which ultimately results in a waste of computing resources or non-convergence of the computing results. Therefore, geometric reconstruction of human tissues is required. In the reconstruction process, the obtained tissue cloud points are first imported into the geometric entity reconstruction software (Geomagic Studio2020) to obtain the constructed tissue cavity. After removing the sharp parts and singular points, the solid model was established from the cavity model to obtain the geometric entity of the tissue. In entity construction, it is necessary to increase the thickness of some thinner tissues to prevent difficulty in the mesh generation. In entity construction, the skin layer compensation thickness was approximately 1 mm. A flowchart for the 3D reconstruction of the CDA-based CVH is shown in Figure 1.

## 3. Modeling of a Finite Element Model

After obtaining the geometric entity models of various arm tissues, the multiphysical field simulation software COMSOL Multiphysics 5.5 was used to implement electromagnetic field modeling of the arm (Computer parameters: CPU: Intel (R) Core (TM) i7-8750H CPU @ 2.20 GHz; Memory: 96.0 GB; Operating system: 64-bit windows 10) and calculation.

As the arm is a 3D structure, 3D modeling is selected in modeling. Considering that we were studying the diffusion of direct current (DC) and alternating current (AC) signals in the human arm, the current components in the AC/DC module were selected for physical field setting, and modeling and analysis are conducted in the frequency (DC signal) and time domain (AC signal), respectively. After completing the physical field setting, the previously constructed geometric entities were imported into the physical field to complete the construction of the digital arm geometric structure.

After completing the model geometry configuration, the carriers of 1 kHz to 1 MHz are loaded into the model to accurately manifest the electrophysiological characteristics of the human tissues. In combination with the volume conductor theory, the conductivity, and the dielectric constant [50,51] of the tissues are shown in Table 1.

According to the volume conductor theory, the human body does not discharge before the electrical signal is injected, and the total charge density is 0; when the frequency of the stimulation signal is within the 1 kHz–1 MHz frequency range, the electromagnetic characteristics of most tissues in the human body can be regarded as those of a quasi-static electric field [52].

### 3.1. Electric Potential Governing Equation

In a quasi-static electric field, the volume conductor must meet $▽\cdot \vec{J}\approx 0$; $\vec{J}$ is the current density (A·m${}^{-2}$), therefore, the electric potential governing equation can be derived as follows:

(1) $$▽\cdot (-\tilde{\sigma_{k}}\vec{▽}\varphi \left(R\right))\approx 0$$

where $\tilde{\sigma_{k}}$ indicates the complex conductivity of the medium in the k−th layer, and the $\tilde{\sigma_{k}}=\sigma_{k}+i\varepsilon_{0}\varepsilon_{rk}$, k=1,2,3,…,N, *k* is the number of tissues layers, where *N* is the outermost layer, which is the skin. $\sigma_{k}$ is the conductivity of the k-th layer tissue and $\varepsilon_{rk}$ is the relative permittivity of the k−th layer tissue. *R* is the polar coordinate position of a point on the surface, and φ is the induced potential in the conductor.

### 3.2. Injection Current Control

In the neural regulation system with electric stimulation, the DC electrical signal is injected into the system through electrodes, so that the signal source can be given as follows:

$$\begin{array}{c} \frac{\partial \varphi (R)}{\partial R_{s}}=\frac{{\vec{J}}_{impressed}}{\tilde{\sigma }} \end{array}$$

where $R_{s}$ represents the polar coordinates of the irregular surface of the arm model at the places covered by positive and negative electrodes. The current signal is injected into the human body through electrodes, so that the current applied by positive and negative electrodes meets the following requirements:

(2) $$\begin{array}{c} {\vec{J}}_{impressed}=\left\{\begin{array}{cc} j, & Electrode(+)\\ 0, & Others\\ -j, & Electrode(-) \end{array}\right. \end{array}$$

where *j* represents the current density (*A*/m${}^{2}$) injected by the stimulation electrode, and $j=\frac{I}{s}$. In the DC electrical signal stimulation system, the injected current will not change with time and it is a constant, that is, I=constant indicates the current intensity (*A*) injected into the electrode; in the AC electrical signal stimulation system, the input current changes with time and is a sine wave signal, so I=Asin(2πft) indicates the current intensity (*A*) injected into the electrode.

Here *s* represents the contact area between electrodes and the model (m${}^{2}$). According to Seo H. et al. and Grossman et al. [47,48], in order to avoid damage to human tissues and prevent patients from obvious tingling, the current range is set as I≤20 mA [53,54]; *s* is the area of the electrode sheet (20 × 20 mm).

### 3.3. Boundary Assumption of the Surface Current

On the organism surface, there is no current signal in the normal direction of the surface at any position except for the places covered by electrodes [54,55], so that the boundary conditions of the model surface can be derived as follows:

$$\begin{array}{c} \frac{\partial \varphi (R)}{\partial R_{\left(S-s\right)}}=0 \end{array}$$

where *S* represents the overall surface of the simplified arm model, and *s* represents the surface of the arm model at the places covered by electrodes.

### 3.4. Mesh Generation

Before model calculation, it is necessary to mesh the model. Considering that there are small or thin areas (≤1 mm) in some (skin, fat, and phalanges) reconstructed tissues, the conventional mesh generation method cannot be completed. In order to ensure the robustness of model calculation and adequate computing resources during model calculation, free tetrahedron meshes were selected for model calculation. Specific parameters are included in Table 2.

## 4. Results

After model boundary setting and mesh generation, the electric field distribution of two different stimulation signals in the tissue was calculated under the DC and AC signal stimulation modes.

**Figure 2:**
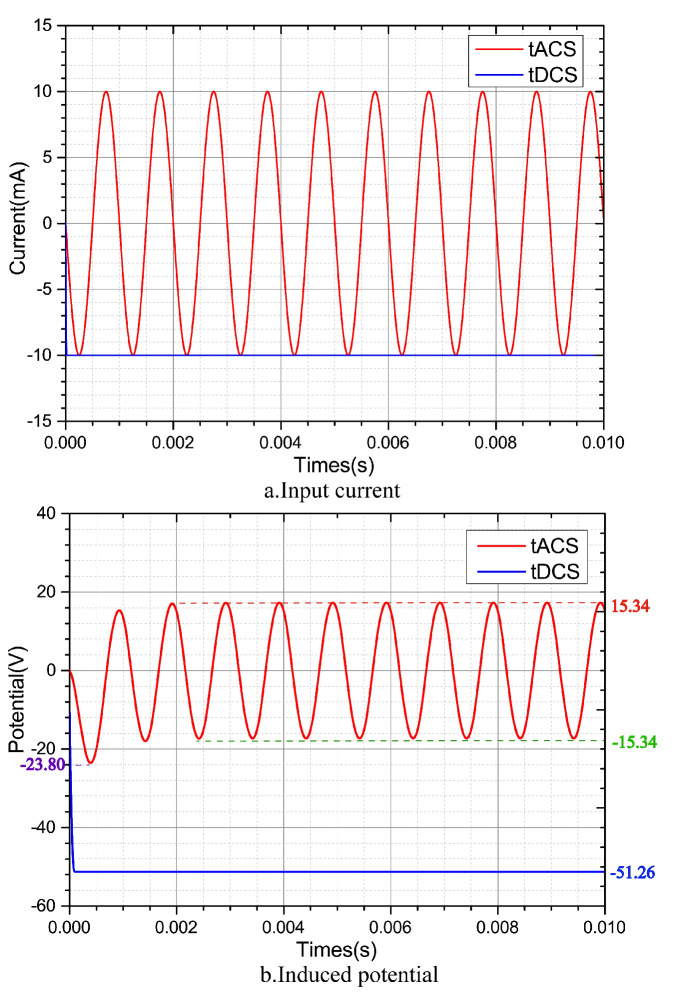
Input current signal and induced coupling potential. (**a**) The current signal waveform injected into the body through electrodes. (**b**) The strength of the coupling potential at the contact surface between the electrode and the body.

According to the DC and AC electrical stimulation characteristics, and considering human safety, a 10 mA DC electrical signal and an AC electrical signal with an amplitude of 10 mA and a signal frequency of 1 kHz were injected into the model (see Figure 2). To analyze the electrical signal changes in the body under the two modes, in terms of tissue electrical characteristics, the carrier frequency of the DC electrical signal was 0, which complicates the model calculation and comparison. Therefore, in model calculation, 1 kHz was used for both DC and AC signal stimulation.

Signal acquisition points were set at the contact point between the electrode center and the skin, and the induced potential change was analyzed by extracting the model calculation results. Figure 2b indicates that in two different stimulation modes, the DC electric stimulation system continuously injected 10 mA current signal into the tissues, which caused the induced potential at the electrodes and the tissues to rise rapidly to 51.26 V, exceeding the human safety voltage of 36 V. Except for 23.8 V in the first cycle, the induced potential of the AC electrical stimulation system was 15.34 V after stable signal transmission, which was within the safe voltage range for the human body.

**Figure 3:**
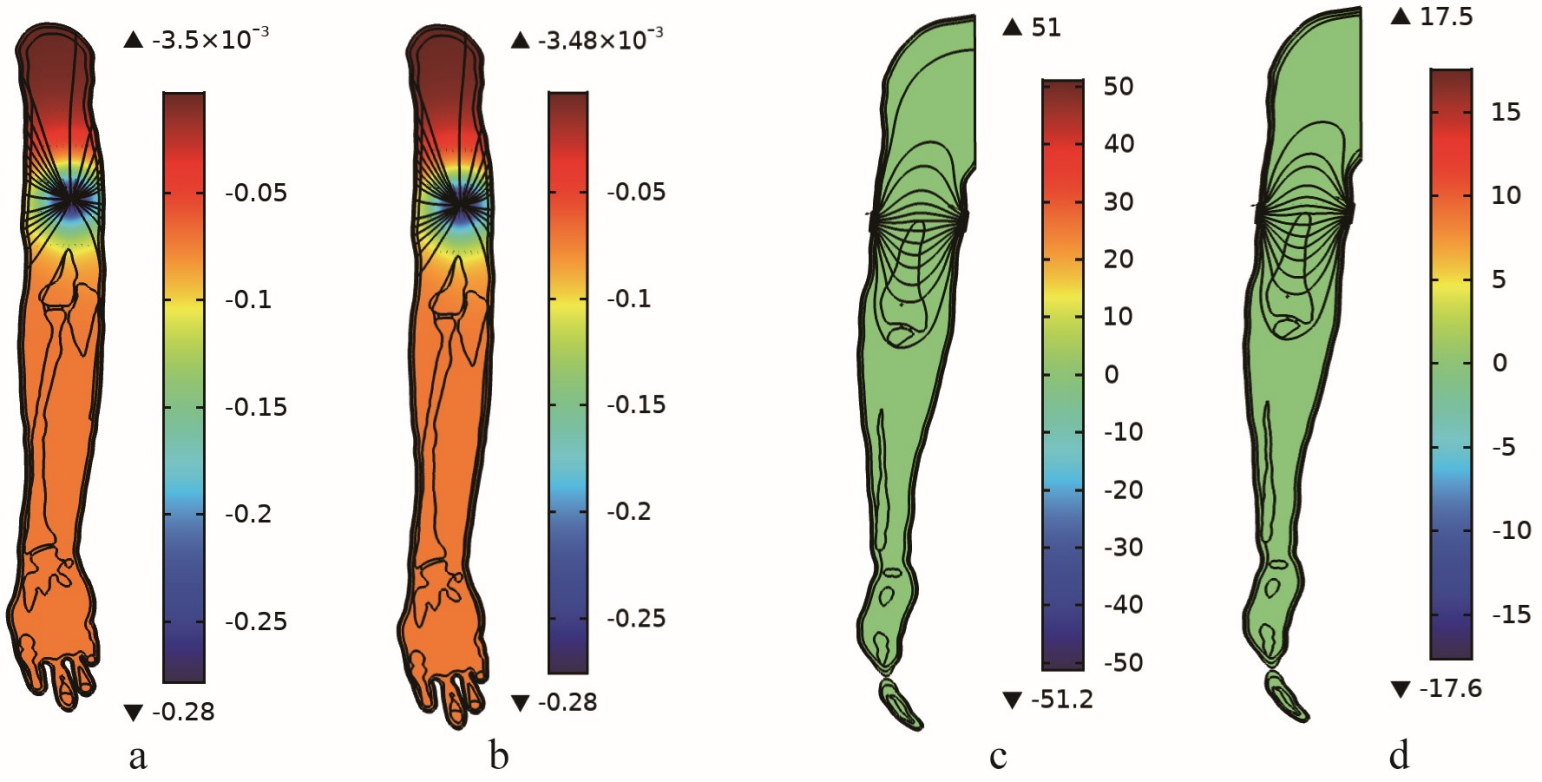
Electric potential distribution diagram of the axial section. (**a**,**b**) Side view sections. (**c**,**d**) Front view sections. (**a**,**c**) Coupling electric potential distribution on the DC electric stimulation section. (**b**,**d**) Coupling electric potential distribution on the AC electric stimulation section.

The two types of axial sections revealed that the difference in potential distribution within the body is relatively small (Figure 3a,b). The coupling potential generated by DC electrical stimulation was considerably stronger than that generated by AC electrical stimulation at the contact between the electrode and the skin (Figure 3c,d), causing the induced potential of the DC electrical stimulation system to exceed the safe voltage of human body, leading to burns.

**Figure 4:**
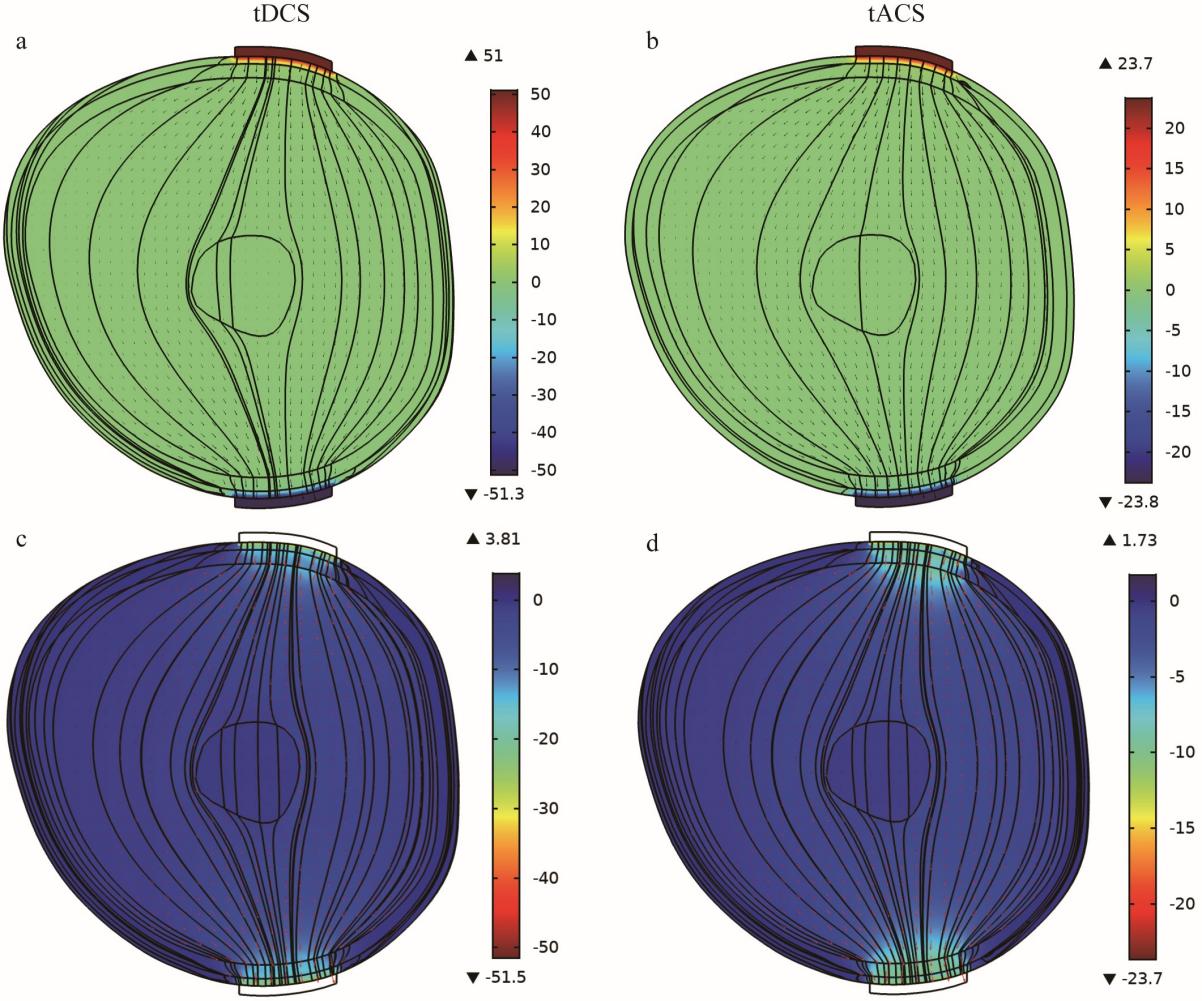
Potential distribution diagram on the cross section. (**a**,**b**) Coupling potential distribution of DC and AC stimulation on the cross section, respectively. (**c**,**d**) Current density distribution of DC and AC stimulation on the cross section, respectively.

To further analyze the effective penetration ability of the signals in the two stimulation modes at any time, the section of the line connecting the electrodes were considered at the observation surface. When $t=4\times 10^{-4}$ s, the distribution of induced potential and current inside the arm is as follows (Figure 4).

Figure 4a,b illustrate that the strongest coupling potential area remains where the electrode makes contact with the skin. However, with DC electric stimulation, the injected current signal is time invariant and a constant, which leads to more notable potential accumulation of the DC electric stimulation system, and the coupling potential also exceeds the human safety voltage. In the AC electrical stimulation system, because the injected current changes with time, and there is a switch between positive and negative electrodes every half cycle, the electric potential stimulation is slightly reduced, and the coupling potential is within the human safety voltage range. Simultaneously, by analyzing the current density distribution in the arm (Figure 4c,d), we observed that the penetration ability of the AC electrical stimulation signal is better than that of DC electrical stimulation signal. When deep electrical stimulation is implemented, the effective depth that the AC electrical stimulation reaches will be greater.

**Figure 5:**
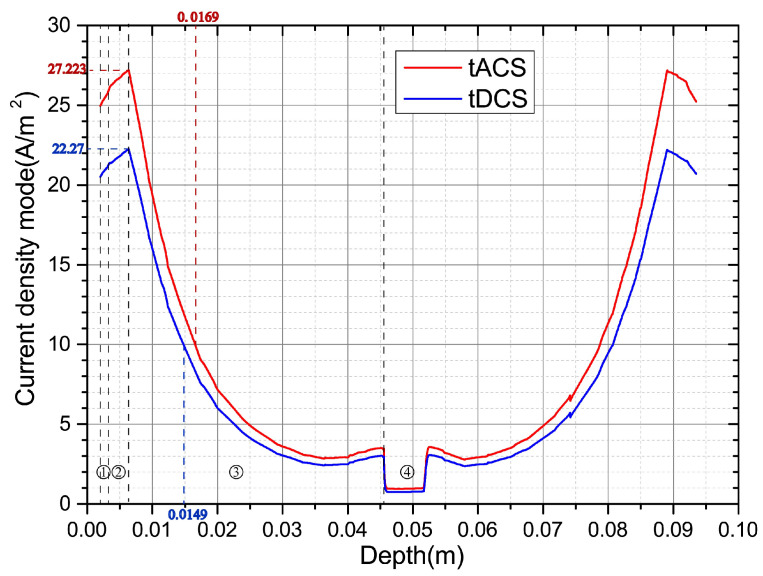
Current density distribution on the geometric center line of electrodes. ①, ②, ③, and ④ illustrate the approximate current density distribution in the skin, fat, muscle, and bone layer, respectively.

The current density along the geometric center line of the two electrodes was extracted for analysis to intuitively analyze the distribution of signal current density on the section (Figure 5).The red line is the distribution of the tACS current density, and the blue line is the distribution of the tDCS current density. At the contact between the electrode and the skin, the coupling current density generated by tACS is 24.96 A/m${}^{2}$, while the coupling current density generated by tDCS is 20.51 A/m${}^{2}$, and the coupling current density of tACS is 21.7% higher than that of tDCS. At the contact of the skin layer ① and the fat layer ②, the coupling current density generated by tACS is 25.72 A/m${}^{2}$, while the coupling current density generated by tDCS is 21.12 A/m${}^{2}$, and the coupling current density of tACS is 21.8% higher than that of tDCS. At the contact of the fat layer ② and the muscle layer ③, the coupling current density generated by tACS is 27.223 A/m${}^{2}$, while the coupling current density generated by tDCS is 22.27 A/m${}^{2}$, and the coupling current density of tACS is 22.2% higher than that of tDCS. In cortical stimulation, the effect of tACS is significantly better than that of tDCS. At the same time, due to the good diffusivity of the muscle layer, the current signal diffuses rapidly in the muscle layer, and the current density is attenuated rapidly. At the muscle and the bone, the coupling current density of tACS is attenuated to 0.96 A/m${}^{2}$, while the coupling current density of tDCS is attenuated to 0.76 A/m${}^{2}$, both of which are attenuated nearly 30 times. In the process of electrical stimulation, assuming that the effective current density of the targeting is greater than or equal to 10 A/m${}^{2}$, the maximum depth that tDCS can reach is 14.9 mm, while tACS can reach 16.9 mm, and the 2 mm difference in effective depth can determine whether it acts on the neural targeting. In the body, the induced current intensity of AC stimulation is higher than that of DC stimulation. Simultaneously, under the equivalent intensity, the depth of AC stimulation is also better than that of DC stimulation. Therefore, when deep electrical stimulation is applied to the body, the system with the AC stimulation signal is better than the system with DC stimulation signal.

## 5. Discussion

**Figure 6:**
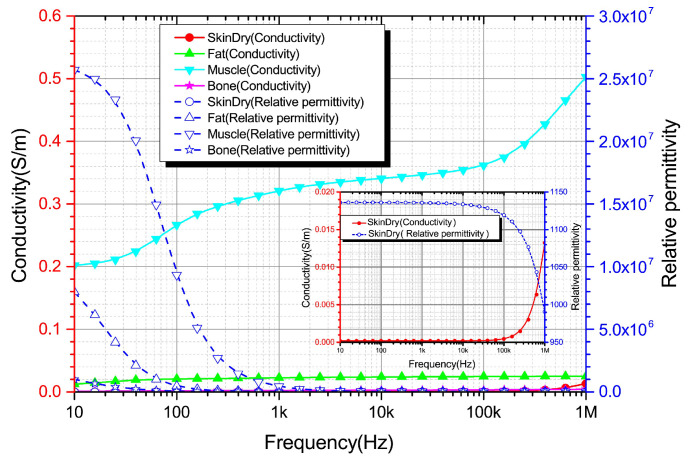
Conductivity and relative permittivity of the human tissues [50,51].

In the present study, to compare the diffusion of the two current signals in the human body, we set the same electrical parameter for the digital arm tissue under DC and AC electrical signal stimulation modes (10 kHz). In fact, according to Gabriel C. et al. and Gabriel S. et al. [50,51], in the ultralow frequency environment, the conductivity of the tissue is lower (Figure 6), and the tissue impedance is larger, so that the coupling potential that is generated is larger, especially since the resistance effect of the skin tissue is more notable. Therefore, in the low-frequency environment, the coupling potential generated at the contact between the skin and the electrode will be larger.

Furthermore, a static field is set, and the effects of living factors such as blood flow and heat scattering are disregarded in the model. However, this may introduce some errors into the model. In future research, we will combine the concept of simplifying blood vessel modeling [56] and tissue segmentation and reconstruction to supplement the influence of blood vessel and blood fluid dynamics in the semirefined model, to make the model more accurate.

**Figure A1:**
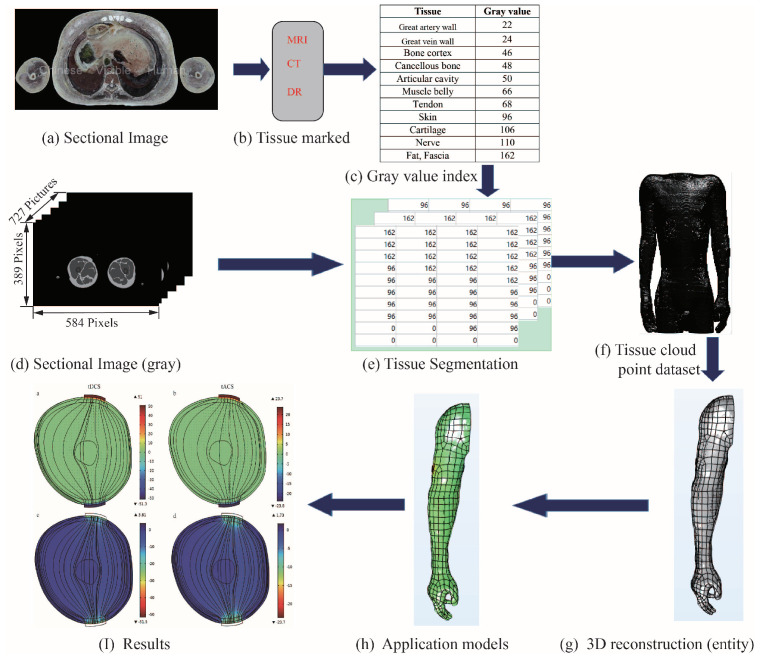
Chinese digital arm production flowchart.

In the present study, only the diffusion of electric signals of the arm was considered. Therefore, in the modeling, we only selected 727 of 1756 human data images containing complete arm data as the data source. In addition, in order to simplify the model, we only selected the right arm as the modeling object, and the latter model expansion (left hand, two legs) and fully refined modeling can refer to the table in the Appendix A, Table A1 to complete the model expansion. For the chest and head, a more complete total grayscale index can be used for segmentation, and the segmentation and reconstruction details can be completed according to the data processing scheme and the process in the Appendix A, Figure A1.

With the application of depth learning in identification [57], in addition to segmentation [58] of images and reconstruction [59,60], in future research, we will focus on combining the depth learning technology to further improve the accuracy of the model. In addition, we will utilize big data technologies to build the nonlinear mapping relationship between [61] stimulation parameters and stimulation effects to provide more comprehensive guidance for stimulation.

Due to some limitations in regard to current density extraction inside the human body, the consistency between the model and the experiment is not discussed in this paper. In future studies, we will design our own hardware system to implement the extraction and verify the consistency between the model and the experiment.

In summary, this model can more truly reflect the non-uniformity of tissue distribution in the human body compared with the uniform simplified model [44,45]. It also demonstrates the performance of electrical stimulation (tDCS, tACS) signals in the human body. Although a series of simplifications have been made in this model, for example, blood vessels [57] and nerves are not described, as they can be depicted by fine modeling. Building the nonlinear mapping relationship from deep learning could be another solution [59,60,61]. Although the extraction of the weak current is limited by special sensors, we are currently trying to demonstrate it by investigating the mechanisms that lead to changes of the induced electric field.

## 6. Conclusions

Digital medical technologies are vital auxiliaries in modern medicine. Through image acquisition, segmentation, individual reconstruction, and finally, inverse engineering processing, the actual entity projection of human tissues can be obtained, which, in turn, allows for the geometric shapes and structures of individual tissues to be projected rapidly to computers, providing personalized references for digital diagnosis, surgery, and treatment, in addition to precision medicine. The present study focused on the electrical stimulation of the arm using the Chinese digital human image dataset as the data foundation. Through image segmentation, tissue reconstruction, and finally, reverse engineering processing, a semirefined digital arm entity model was obtained. To analyze the current signal distribution in the human body under two stimulation modes, tDCS and tACS, we constructed transient models of the two modes. Through model calculation, we established that DC electrical stimulation was more prone to high-voltage burns, providing a reference for the selection of the electrical stimulation mode. Simultaneously, the model can also be used to analyze signal diffusion and tissue damage inside the arm in other physical fields (physics, chemistry, biology, etc.). Such findings are especially significant for the analysis of factors with difficult detection inside the body, and may provide a vital theoretical basis for future studies.

## Figures and Tables

**Table 1:** The tissue’s electrical parameters.

Tissue	Frequency	Conductivity	Relative
	(Hz)	σ (S/m)	Permittivity $\varepsilon_{r}$
	1 k	2.00 × $10^{-4}$	1.14 × $10^{3}$
Skin	10 k	2.04 × $10^{-4}$	1.13 × $10^{3}$
	100 k	4.51 × $10^{-4}$	1.12 × $10^{3}$
	1 M	1.32 × $10^{-2}$	9.91 × $10^{2}$
	1 k	2.24 × $10^{-2}$	2.41 × $10^{4}$
Fat	10 k	2.38 × $10^{-2}$	1.09 × $10^{3}$
	100 k	2.44 × $10^{-2}$	9.29 × 10
	1 M	2.51 × $10^{-2}$	2.72 × 10
	1 k	3.20 × $10^{-1}$	4.30 × $10^{5}$
Muscle	10 k	3.40 × $10^{-1}$	2.60 × $10^{4}$
	100 k	3.60 × $10^{-1}$	8.10 × $10^{3}$
	1 M	5.00 × $10^{-1}$	1.80 × $10^{3}$
	1 k	2.02 × $10^{-3}$	2.70 × $10^{3}$
Skeleton	10 k	2.04 × $10^{-3}$	5.22 × $10^{2}$
	100 k	2.08 × $10^{-3}$	2.27 × $10^{2}$
	1 M	2.44 × $10^{-3}$	1.45 × $10^{2}$

**Table 2:** Mesh Parameters.

Parameter Type	Mesh Setting		Mesh Parameters		
	**Sequence**	**Cell**	**Domain**	**Boundary**	**Edge**
	**Type**	**Size**	**Cell**	**Element**	**Cell**
Parameter	User control mesh	More refined	893,031	213,151	36,774

## Data Availability

Not applicable.

## Abbreviations

The following abbreviations are used in this manuscript:

CDA	Chinese Digital Arm
tDCS	transcranial Direct Current Stimulation
tACS	transcranial Alternating Current Stimulation
CVH	Chinese Visible Human

## Appendix A

### Appendix A.1. Chinese Digital Arm Production Flowchart

Shown in Figure A1 below.

### Appendix A.2. Image Gray Threshold

Table A1 shows the gray values of all the segmented tissues after multimodal marking in this paper. In the process of segmentation in the present study, only the influence of the main human tissues on signal transmission is considered at present; therefore, in the process of MIMICS tissue segmentation, the first step is to set the threshold value to 22–162, build a full-tissue master mask, and effectively remove the influence of the background. The second step is to adjust the threshold to 96 to extract the skin tissue. The third step is to set the threshold to 162 to extract the adipose tissue. The fourth step is to adjust the threshold to 46–48 to extract the bone tissue. After performing Boolean operations on the extracted tissues and the master, all the remaining tissues are defined as muscle tissues (including blood vessel wall, muscle, nerve, and cartilage), so as to achieve tissue segmentation.

**Table A1:** Tissue’s Gray Values..

Tissue	Gray Value	Tissue	Gray Value
Aorta wall	22	Muscle tendon	68
Vena cava	24	Skin	96
Bone cortex	46	Cartilage	106
Cancellous Bone	48	Nerve	110
Articular cavitye	50	Fat	162
Muscle belly	66		

### Appendix A.3. Math Symbol Notes

Shown in Table A2 below.

**Table A2:** Math symbols.

Math Symbols	Unit	Note
$\vec{J}$	A·m${}^{-2}$	Current density
$\tilde{\sigma_{k}}$		Complex conductivity
σ	(S/m)	Conductivity
$\varepsilon_{r}$		Relative permittivity
φ	V	Induced potential
${\vec{J}}_{impressed}$	A·m${}^{-2}$	Injected Current density
*S*	m${}^{2}$	Overall surface area
*s*	m${}^{2}$	Electrode area (20 × 20 mm)
*I*	A	Injected Current
